# Polymer-Supported Heterogeneous Fenton Catalysts for the Environmental Remediation of Wastewater

**DOI:** 10.3390/molecules29102188

**Published:** 2024-05-08

**Authors:** Bakhta Bouzayani, Maria Ángeles Sanromán

**Affiliations:** 1 Laboratory of Physical Chemistry of the Solid State, Department of Chemical, University of Sfax, Sfax 3000, Tunisia; bbouzayanibakhta@gmail.com; 2 CINTECX, Department of Chemical Engineering, University of Vigo, Campus As Lagoas-Marcosende, 36310 Vigo, Spain

**Keywords:** polymeric supports, heterogeneous Fenton catalysts, environmental remediation, wastewater treatment

## Abstract

Materials based on polymer hydrogels have demonstrated potential as innovative Fenton catalysts for treating water. However, developing these polymer-supported catalysts with robust stability presents a significant challenge. This paper explores the development and application of polymer-supported heterogeneous Fenton catalysts for the environmental remediation of wastewater, emphasizing the enhancement of metal incorporation into catalysts for improved efficiency. The study begins with an introduction to the heterogeneous Fenton process and its relevance to wastewater treatment. It further delves into the specifics of polymer-supported heterogeneous Fenton catalysts, focusing on iron oxide, copper complexes/nanoparticles, and ruthenium as key components. The synthesis methods employed to prepare these catalysts are discussed, highlighting the innovative approaches to achieve substantial metal incorporation. Operational parameters such as catalyst dosage, pollutant concentration, and the effect of pH on the process efficiency are thoroughly examined. The catalytic performance is evaluated, providing insights into the effectiveness of these catalysts in degrading pollutants. Recent developments in the field are reviewed, showcasing advancements in catalyst design and application. The study also addresses the stability and reusability of polymer-supported heterogeneous Fenton catalysts, critical factors for their practical application in environmental remediation. Environmental applications are explored, demonstrating the potential of these catalysts in addressing various pollutants. The Conclusions offers future perspectives, underlining the ongoing challenges and opportunities in the field, and the importance of further research to enhance the efficacy and sustainability of polymer-supported heterogeneous Fenton catalysts for wastewater treatment.

## 1. Introduction

The growth of diverse industries, propelled by technological advancements, has led to an escalation of pollution. Various organic pollutants stemming from industrial sources, like leather, paint, and textiles, disseminate into the water and environment, leading to adverse consequences [1,2,3]. These pollutants such as polycyclic aromatic hydrocarbons, dyes, antibiotics, phenolic compounds, herbicides, …, are highly toxic and, consequently, pose a threat to both humans and the ecosystem [4,5,6]. Presently, organic pollutants have garnered attention due to their adverse impact on aquatic systems and human health coupled with the difficulty in eliminating and eradicating them from the environment [7,8,9]. These pollutants often exhibit properties such as water solubility and limited biodegradability, presenting significant challenges for disposal. Disposing of these contaminants is a significant and contentious challenge for scholars since some exhibit low reactivity and contain intricate compounds [10,11]. One of the crucial duties of reducing environmental contamination is to select a method that is both effective and environmentally friendly. There are various methods for eliminating organic contaminants, such as chemical, physical, and biological techniques. Nevertheless, they are ineffective and impractical due to drawbacks like the generation of hazardous by-products, high costs, and energy consumption [12].

In the past few years, advanced oxidation processes (AOPs) have been explored as a potential method for treating organically contaminated water, counting on the in situ production of hydroxyl radicals (${HO}^{•}$), possessing a robust oxidation capacity (standard potential = 2.80 V versus standard hydrogen electrode) [13,14]. During AOP treatment, complex organic compounds can potentially be oxidized by generated ${HO}^{•}$ toward smaller organic compounds or fully mineralized to carbon dioxide (${CO}_{2}$) and $H_{2}O$ [15,16,17]. Among the multitude of AOPs, the processes based on the Fenton reaction exhibit outstanding decontamination capabilities due to the production of ${HO}^{•}$ throughout the procedure, where ${Fe}^{2+}$ is employed as the catalyst and hydrogen peroxide ($H_{2}O_{2}$) as the oxidant. The overall process of the Fenton mechanism may be represented as described in Equations (1)–(3):

(1) $$\;{Fe}^{2+}+H_{2}O_{2}\;\;\to \;{Fe}^{3+}+{OH}^{-}+{HO}^{•}$$

(2) $${Fe}^{3+}+H_{2}O_{2}\;\to \;{Fe}^{2+}+{HO}_{2}^{•}+H^{+}$$

(3) $$Organic\;matter\;\;\;+{HO}^{•}\;\to degradation\;products$$

The reaction between hydrogen peroxide and ${Fe}^{2+}$ leads to the formation of ${HO}^{•}$. Nonetheless, recovering ${Fe}^{2+}$ (Equation (2)) poses a challenge due to the intrinsically slow kinetics of ${Fe}^{3+}$ to ${Fe}^{2+}$ reduction [18].

The Fenton process offers numerous benefits, including excellent performance and simplicity, because it operates at atmospheric pressure and room temperature for the oxidation of organic compounds, and its non-toxicity. Considering the aforementioned benefits, the Fenton procedure has been applied to address various types of wastewaters like olive press wastewaters [19], reactive dyes [20], pesticides [21,22], cosmetics [23], dyes [24,25], pharmaceuticals [26,27], cork cooking [28], pulp mill liquid wastes [29], and chloro-substituted phenols [30]. However, notwithstanding the extensive research on the Fenton process and its effective performance in the mentioned industrial water remediation, the traditional Fenton process still has drawbacks that impede its industrial application. Fenton oxidation is highly affected by the pH of the solution, requiring it to be kept in an acidic pH range (consistently operates most efficiently at pH around 3) to prevent the precipitation of ${Fe}^{3+}$ into iron hydroxide. The difficulty of working in acidic conditions and precise pH control hampers the practical application of the Fenton reaction. An increase in pH provokes precipitation of iron oxide, leading to excessive sludge production. As a result, the substantial volume of iron sludge generated represents an additional shortcoming of Fenton oxidation. These sludges pose a risk of secondary pollution and need treatment. The need for additional sludge treatment can add complexity and operational costs to the treatment process. A further limitation of the Fenton reaction is its high chemical consumption, which remarkably limits its practical applications [31]. To surmount these limitations, researchers have focused on improving the Fenton process by the use of heterogeneous catalysts to replace ${Fe}^{2+}$, for example, iron minerals such as ${Fe}_{2}O_{3}$ [32], ${Fe}_{3}O_{4}$ [33], FeOOH [34], pyrite [35], zero-valent iron (ZVI) [36], or a multi-metal compound (e.g., layered double hydroxide) [37], or by immobilization into a solid matrix such as a zeolite [38], carbon material [39], polymers [40], etc. An exemplary heterogeneous Fenton catalyst should exhibit characteristics including reactivity on par with the homogeneous Fenton process, cost-effectiveness, wide-ranging pH activity, exceptional stability with minimal iron leaching, and easy separability [41]. In addition, the excellent heterogeneous catalysts function as reformers, decomposing $H_{2}O_{2}$ on their surface and employing $H_{2}O_{2}$ as the only reactant. This approach yields the benefit of preventing precipitation of iron hydroxides and leaching, and facilitates operation in flow systems. The sustained performance of the catalysts ensures effective Fenton activity across a broad range of pH. These well-established approaches are known as heterogeneous Fenton-like processes.

In recent years, the use of diverse heterogeneous catalysts with enhanced performance has been proposed by several publications. As was mentioned above, these can be broadly classified into two main types: independent catalysts (including ZVI, metallic minerals, iron oxides/hydroxides, or multi-metallic catalysts) and catalysts immobilized into materials such as clays, zeolites, and polymers. Using polymers as supports for heterogeneous Fenton catalysts appears to be a promising approach for water treatment, leveraging the widespread use of polymers due to their advantageous characteristics such as high strength, lightweight design, and cost-effectiveness.

This review aims to provide a summary of the latest polymer-supported iron, copper, and ruthenium-based catalysts for Fenton-like reactions and their synthesis strategies. The influencing factors in the Fenton reaction and the evaluation of catalytic performance are discussed. This study strives to provide an overview of the development of polymer-supported heterogeneous catalysts for the elimination of recalcitrant organics in wastewater through the Fenton reaction. The stability and reusability of polymer-supported heterogeneous Fenton catalysts are also addressed, and potential environmental applications are introduced. The conclusions and future perspectives are also covered.

## 2. Polymer-Supported Heterogeneous Fenton Catalysts

Polymers loaded with iron oxide or copper have commonly been used as heterogeneous Fenton catalysts to facilitate the presence of a Fenton-like mixture in a solution and to overcome the limitations of conventional Fenton processes. In recent years, numerous publications have suggested employing diverse varieties of polymer-supported heterogeneous Fenton catalysts. Polymer carriers can be grouped into synthetic, semi-synthetic, or biopolymers depending on their source. Among these classes, synthetic and biopolymers have found extensive application as catalyst supports for heterogeneous Fenton-like reactions. In these instances, the properties and characteristics of the polymer play a pivotal role in modulating activity at catalytic sites [42]. Therefore, an ideal supporting material should exhibit the following properties: high surface area, chemical inertness towards various harsh conditions, and surface functionality.

Polymeric material can act as a shield to protect the catalytic compounds from excessive leaching and mechanical corrosion, thereby improving their durability. In addition, this section describes several innovative techniques for immobilizing heterogeneous Fenton catalysts within polymeric supports, such as coating the catalyst onto polymer surfaces using the method of layer-by-layer assembly [43]. Furthermore, another technique which is as significant as the first entails crosslinking the heterogeneous Fenton catalyst with the polymeric material using crosslinking agents; this leads to anchoring the catalyst within the polymer matrices [44]. In this regard, various crosslinking agents are employed such as glutaraldehyde [45], acrylate [46], and polyethylene glycol [47]. The third method is the incorporation of iron or iron oxide as a heterogeneous Fenton catalyst directly during the synthesis of the polymer. This can be achieved through copolymerization, ensuring that the catalyst is evenly distributed throughout the polymer matrix and enabling strong interaction between the components, which is crucial for its catalytic activity [48]. There is also another way, which is microencapsulation, consisting of encapsulating heterogeneous Fenton catalysts in polymer capsules. This technique guards the catalyst from harsh environments and enhances its stability [49]. The electrospinning technique is also considered very important for immobilizing iron or iron oxides in polymers, given its frequent adoption in recent years [50]. These techniques offer diverse strategies for immobilizing Fenton catalysts within polymeric supports and allow for the straightforward retrieval and recycling of catalysts. Various synthetic polymers serve as support materials to enhance the stability and efficiency of Fenton-like reactions such as polyvinyl alcohol (PVA) [51], polyethylene (PE) [52], and poly(3,4-ethylene-dioxythiophene) (PEDOT) [53]. The biopolymers frequently employed as carriers for heterogeneous Fenton catalysts include alginate [54], pullulan [55], chitosan [56], starch [57], and cellulose [58]. They are commonly preferred as supports because of their specific properties that make them suitable for catalytic Fenton-like reactions.

### 2.1. Iron-Based Catalysts

Iron-based catalysts, in particular iron oxides and iron-containing compounds, have demonstrated remarkable catalytic activity in various AOPs for the degradation of organic pollutants. Iron oxides constitute the most prevalent materials in the Earth’s crust [59]. Among the iron oxides extensively employed in environmental remediation processes are magnetite (${Fe}_{3}O_{4}$), hematite (α${Fe}_{2}O_{3}$), maghemite (γ ${Fe}_{2}O_{3}$), and goethite (αFeOOH).

Iron oxide nanomaterials (FeO NMs) exhibit a specific affinity for diverse environmental remediation purposes and offer a substantial surface area [60,61,62,63]. Due to its properties, including its extremely small size and surface modifiability, the utilization of iron oxide has garnered significant attention [64,65]. FeO NMs commonly employ the Fenton reaction. However, unmodified FeO NMs face certain limitations, including iron leaching in acidic conditions and a decline in catalytic activity for organic degradation at pH levels exceeding 3 [66]. The primary limitation of iron oxide in the Fenton reaction is its susceptibility to agglomeration. Therefore, to address these limitations considerable efforts have been invested in developing innovative materials. The incorporation of FeO NMs into a supporting material imparts superior properties to the composite, thereby heightening the catalytic efficiency. Iron oxide-supporting polymers [67,68,69,70] have been described. The integration of iron oxide nanoparticles (NPs) within a polymer matrix enhances the stability and efficiency of the catalyst. This composite material demonstrates promising catalytic activity in the decomposition of hydrogen peroxide, generating ${HO}^{•}$ crucial for the degradation of harmful substances in wastewater [71,72]. Polymer supports provide additional benefits such as increased surface area, improved recyclability, and reduced metal leaching, making them valuable tools in environmental remediation processes [73,74].

PEDOT has been utilized as an outer layer in core-shell NPs, enhancing active sites and protecting iron oxide in Fenton reactions. Shin et al. [53] synthesized ${Fe}_{3}O_{4}$–PEDOT core-shell NPs by acid-etching-mediated chemical oxidation polymerization for the removal of Reactive Black 5 (RB5) and Orange II, exhibiting approximately 2.5 times higher catalytic activity compared to commercial ${Fe}_{3}O_{4}$ nanopowder [53]. González-Bahamón et al. [75] prepared Fe–PE film for heterogeneous Fenton oxidative degradation using resorcinol as the target pollutant. With this material, at a non-adjusted initial pH of 5.6 in the presence of $H_{2}O_{2}$, total degradation of resorcinol was observed in 40 min. Ratvijitvech et al. [76] developed a catechol-based hypercrosslinked polymer (catechol-HCP) as a low-cost solid support for iron (catechol-HCP-Fe) and used like a heterogeneous Fenton catalyst for the degradation of methylene blue (MB). The decoloration of 100 ppm MB occurred within 25 min.

**Figure 1:**
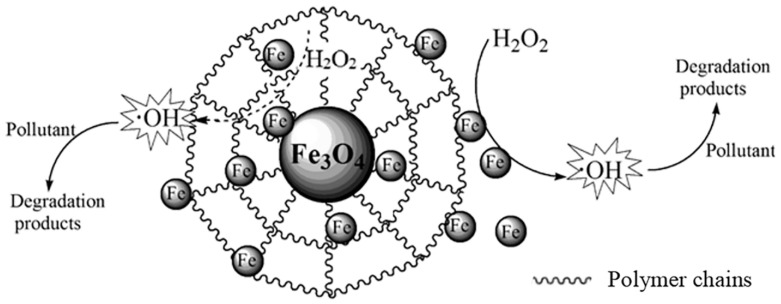
Suggested mechanism for the removal of pollutants by polymer-incorporated ${Fe}_{3}O_{4}$ in a Fenton-like system. Reproduced from [77]. Copyright 2019 with permission from Spinger Nature (Berlin, Germany).

Additionally, alongside synthetic polymers, biopolymers are also utilized as supports for Fenton catalysts. Several biopolymers have been employed to incorporate iron into their matrices. Thus, in Figure 1, the catalyst mechanism described by Shen et al. [77] in a Fenton-like process is presented. The good results obtained can be explained based on the exceptional hydrophilicity, permeability, and mass transfer efficiency of polymeric materials such as hydrogels. These properties facilitate the interfacial reaction between the oxidant, the catalyst, and the contaminants of interest.

Alginate has garnered the interest of researchers due to its ability to produce stable hydrogel spheres. It can act as a catalytic support owing to its multiple positive features including high surface area, low cost, environmental friendliness, network structure, and large amount of surface groups [78]. The ionization of hydroxyl groups in an aqueous solution results in the negative charge of polymeric alginate chains, inducing alginate to undergo ion exchange and form multivalent cations [79]. These structures create an insoluble framework and are described by the egg-box model [80].

In this context, the research conducted by Sanroman’s team on an alginate-supported Fenton catalyst is noteworthy. They have utilized this heterogeneous catalyst for the remediation of contaminants like pesticides [81], winery wastewater [82], ionic liquids [83], and dyes [84]. Titouhi et al. [85] synthesized iron-incorporated alginate beads (Fe-ABs) and used them as a heterogeneous catalyst for the elimination of ofloxacin via Fenton oxidation. They achieved complete removal of the antibiotic within 180 min, demonstrating minimal iron leaching and maintaining good stability through three successive oxidation processes. Similarly, Ben Hammouda et al. [86] suggested the application of alginate spheres containing iron as a heterogeneous catalyst for degradation of the malodorous compound ‘indole’ through Fenton oxidation. Approximately 82% of indole was removed within a 120 min reaction. Subsequently, they fabricated catalysts using alginate beads based on FeO NMs (Fe-MABs). Fe-MABs were shown to be the best heterogeneous Fenton catalysts for the oxidation of 3-methylindole by $H_{2}O_{2}$.

**Figure 2:**
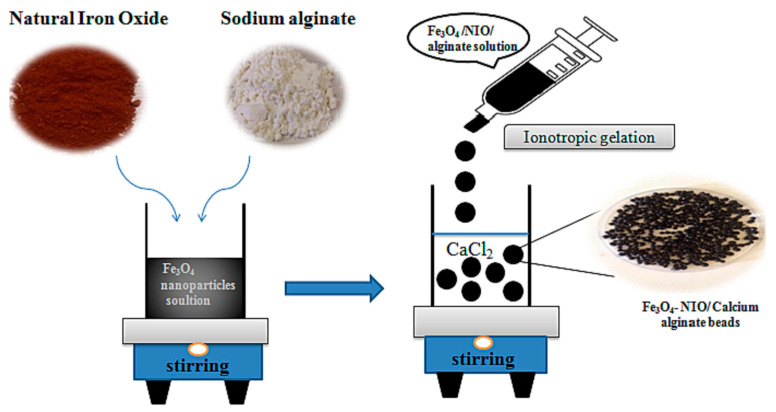
Graphical depiction of the preparation of alginate-bead-supported iron oxide and NIO. Reproduced from [88]. Copyright 2024 with permission from Elsevier (Amsterdam, The Netherlands).

The use of alginate as a polymer support for iron oxide in the Fenton reaction leverages its inherent properties, creating an efficient catalyst for pollutant degradation and aligning with the principles of environmental sustainability [87]. The synthesis procedure is easy, as is shown in Figure 2, where the procedure to obtain magnetic calcium alginate beads in which the FeO and natural iron ore (NIO) are embedded in the hydrogel is described. Natural iron ore improves the stability of the heterogeneous catalyst, makes it stronger, and also increases its degradation activity [88].

**Figure 3:**
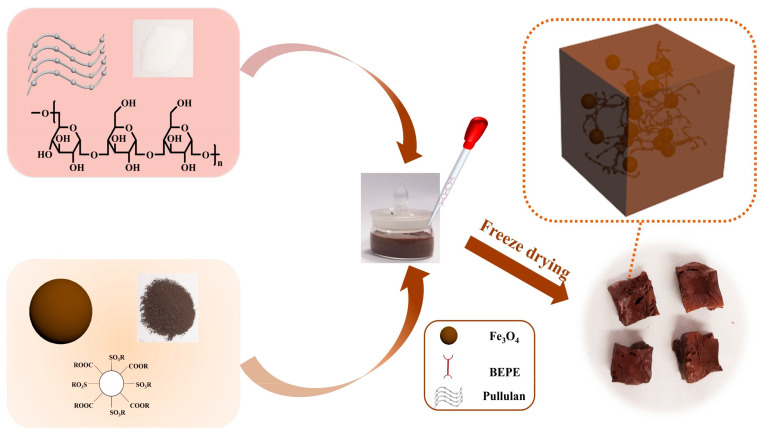
Schematic representation of the fabrication process for magnetic pullulan hydrogels. Reproduced from [55]. Copyright 2024 with permission from Elsevier (Amsterdam, The Netherlands).

Table 1 summarizes several examples of iron oxide/polymer mixed composites. Among them, an interesting hydrogel for the synthesis of catalysts is pullulan, which is a three-dimensional polymeric material formed by the crosslinking of the natural polysaccharide pullulan. These hydrogels are highly hydrophilic and possess a porous structure that allows them to retain large amounts of water within their three-dimensional network. Due to their unique properties, such as biocompatibility, biodegradability, and water absorption capacity, pullulan hydrogels are used in various biomedical applications, such as matrices for controlled drug release, scaffolds for tissue engineering, encapsulation materials in the food industry, and biomaterials for surgical and regenerative applications. In addition, applications have also been found in the field of metal retention. For example, Cheng et al. [55] synthesized magnetic pullulan hydrogels by incorporating ${Fe}_{3}O_{4}$ into pullulan matrices (Figure 3) for the oxidative degradation of the antibiotic tetracycline (TC) as the target contaminant via a heterogeneous Fenton process. The developed catalyst exhibited activity in the presence of hydrogen peroxide, substantially improving the degradation rate.

**Figure 4:**
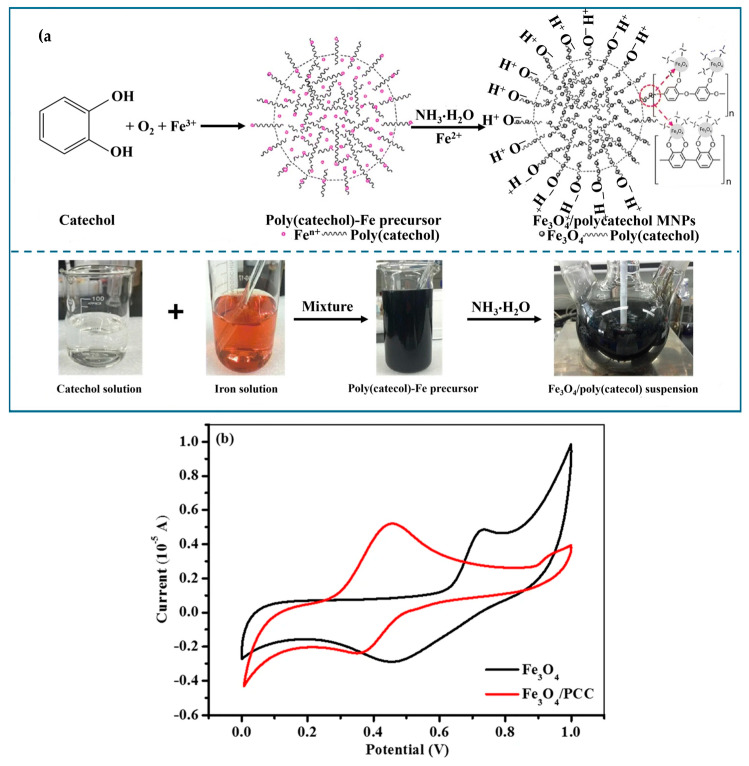
(**a**) Procedure for the synthesis of ${Fe}_{3}O_{4}$/PCC MNPs and (**b**) CV scans with ${Fe}_{3}O_{4}$/PCC- and ${Fe}_{3}O_{4}$-modified glassy carbon electrodes in aqueous solution, ${[Na}_{2}{SO}_{4}$] = 0.1 M and pH = 6. Reproduced from [89]. Copyright 2021 with permission from Springer Nature (Berlin, Germany).

Poly(catechol) is a type of biopolymer derived from the polymerization of catechol, which is a natural compound found in various organisms. For its cohesive and adhesive properties poly(catechol) has gained attention, and it has been explored for diverse applications, such as coating material. The polymerization of catechol can be initiated by various methods, and the resulting poly(catechol) structure exhibits unique characteristics based on its chemical composition and bonding. As depicted in Figure 4a the polymerization of catechol is catalyzed by ferric iron, generating a precipitate containing the poly(catechol)-Fe precursor.

Poly(catechol)-modified ${Fe}_{3}O_{4}$ magnetic nanocomposites (${Fe}_{3}O_{4}$/PCC MNPs) used as heterogeneous catalysts can effectively facilitate the elimination of organic molecules such as MB through the Fenton reaction. Furthermore, ${Fe}_{3}O_{4}$/PCC MNPs can be utilized for eight cycles of MB degradation with minimal iron loss. This study illustrated that poly(catechol) is a promising support for heterogeneous Fenton catalysts [89]. This catalyst exhibited higher efficiency compared to ${Fe}_{3}O_{4}$ in degrading the pollutant. Cyclic voltammetry (CV) studies revealed that the peak potential difference between the cathodic and anodic peaks of ${Fe}_{3}O_{4}$/PCC MNPs was smaller than that of ${Fe}_{3}O_{4}$ MNPs (Figure 4b), indicating an acceleration of the ${Fe}^{3+}/{Fe}^{2+}$ redox processes following the introduction of poly(catechol). The faster regeneration of Fe(II) is highlighted as a significant factor that can greatly enhance the degradation of MB in the heterogeneous Fenton system. This suggests that the presence of PCC on the surface of ${Fe}_{3}O_{4}$ MNPs improves the efficiency of the Fenton reaction, making it more effective in degrading organic pollutants.

The recyclability/reusability of heterogeneous polymer-supported catalysts in Fenton reactions is a crucial parameter due to its economic implications. Eight cycles of the Fenton reaction were performed with the ${Fe}_{3}O_{4}$/PCC MNPs catalyst for the elimination of MB [89]. Throughout the eight recycling iterations, MB was almost eliminated with negligible iron leaching. The minimal iron release (<1.5 mg L^−1^) and stable catalytic efficiency made ${Fe}_{3}O_{4}$/PCC MNPs a desirable catalyst for Fenton reactions.

Zhuang and co-workers evaluated the catalytic efficacy of iron (hydr)oxides in a PVA hydrogel as a heterogeneous Fenton catalyst for TC degradation. The resulting material exhibited good catalytic activity in a pH range from 2 to 10, with low iron leaching and excellent reusability, and it maintained a level of nearly 90% after five consecutive cycles, indicating that the hydrogels presented strong recyclability, maintaining high catalytic activity. In addition, the percentage of leached iron from each catalyst after the reuse experiment was below 5%, further confirming the PVA hydrogel’s effective inhibition of iron ion leaching [92].

In several cases, a combination of polymers was used. Thus, Meijide et al. [91] crafted beads composed of PVA and alginate, incorporating goethite (G-PVA-A) as the heterogeneous catalyst and the source of iron. The catalysts were employed in the elimination of 1-butylpyridinium chloride using the Fenton procedure. Under optimal conditions, the complete removal was attained within 1 h. G-PVA-A remains stable and recyclable, despite a slight reduction in efficacy after three consecutive cycles. Shen and co-workers prepared carboxymethyl cellulose-g-poly(acrylic acid co-acrylic amide) hydrogel-coated FeO NMs by a coprecipitation method followed by an in situ graft copolymerization as heterogeneous catalysts for the degradation of phenol [77]. Under optimal conditions, approximately 80.4% of the chemical oxygen demand (COD) and 98.2% of the phenol were eliminated within 180 min.

Chitosan, a biopolymer derived from chitin, is present in the exoskeletons of crustaceans [90]. It has a distinctive feature that makes it valuable in various applications. The existence of amino and hydroxyl groups on chitosan, coupled with its susceptibility to modification through chemical and physical means, makes this material an appealing support matrix for a range of inorganic nanomaterials such as iron oxide and copper [93]. Chitosan-supported iron oxide emerges as a promising and environmentally compatible option for heterogeneous Fenton catalysis in environmental remediation [94]. This is further enhanced when using magnetic chitosan beads for Fenton mineralization, combining effective pollutant degradation with easy separation and reuse of the catalyst. After the Fenton oxidation process, the recycling of ion salts is an important consideration for environmental and economic sustainability; ion salts, can be recovered using ion exchange resins. These resins selectively capture specific ions from the solution, allowing for their separation and potential reuse. Another method involves the crystallization of salts from the solution through evaporation or cooling processes. This can result in the formation of pure salt crystals that can be collected and reused or processed for other applications. It is essential to consider the composition of the salts and any potential contaminants when planning their recycling. Proper treatment methods should be employed to ensure the purity and quality of recycled salts. Its utilization as a polymer support combined with iron oxide presents numerous advantages as a heterogeneous catalyst. The key advantages of this catalytic system contribute to its potential effectiveness and sustainability. Firstly, the intrinsic properties of chitosan as a biocompatible and biodegradable material align with environmental considerations, ensuring a minimal impact on ecosystems [95]. Li et al. [96] evaluated the catalytic activity of chitosan-supported ${Fe}_{3}O_{4}$ in the degradation reaction of tetracyclines (TCs) via the Fenton reaction. In the chitosan–${Fe}_{3}O_{4}$/$H_{2}O_{2}$ system, the removal rate of TC was significantly accelerated compared to that of the ${Fe}_{3}O_{4}$/$H_{2}O_{2}$ system, with almost 96.0% of TCs being removed within 20 min. On one hand, the use of the chitosan biopolymer as a support effectively prevents the agglomeration of ${Fe}_{3}O_{4}$ NPs. On the other hand, the decomposition of $H_{2}O_{2}$ can be triggered by the robust synergy between Fe-based groups and the carbon matrix, consequently enhancing the degradation efficiency. Subsequently, an ample amount of ${HO}^{•}$ will be liberated, which demonstrates the catalyst’s primary reactive groups’ hetero-catalytic effect in the removal of TC in the latter mechanism analysis.

The high surface area of the chitosan-supported iron oxide catalyst is another notable advantage. This feature provides an increased number of active sites for catalytic reactions, optimizing the efficiency of pollutant degradation in water treatment processes. Moreover, the versatility of chitosan is a valuable attribute [93]. The material can be easily modified, allowing for the customization of the catalyst’s properties to suit specific applications and enhance its overall efficacy. Cost-effectiveness is also a significant advantage of chitosan-supported iron oxide. Derived from natural sources, chitosan contributes to the economic feasibility of the catalyst, making it an accessible and sustainable option for environmental remediation [94]. The ease of recovery is an operational advantage, as the catalyst can be readily separated and recovered from reaction mixtures. This characteristic facilitates its reuse in multiple cycles, contributing to the overall sustainability of the catalytic process [96]. Thus, the high recyclability of magnetite NPs embedded into chitosan beads was confirmed during six cycles in the heterogeneous Fenton-like degradation of TCs [96]. After each cycle, the beads were isolated from the system by imposing an external magnetic field and further freeze-dried for 24 h. In each cycle, TCs and $H_{2}O_{2}$, were consistently introduced with the same concentrations. After six cycles, more than 85% of TCs degraded within 120 min, indicating that it is an effective catalyst for the heterogeneous Fenton process.

Various researchers have provided evidence of the effectiveness of iron-loaded chitosan in the context of heterogeneous Fenton reactions. Thus, a chitosan-supported iron oxide catalyst demonstrates an improved Fenton reaction efficiency for degrading organic pollutants such as phenol, triclosan, and 3-chlorophenol in water [97]. This performance enhancement positions the catalyst as a valuable tool in the arsenal of environmental remediation strategies. The amalgamation of these advantageous features positions chitosan-supported iron oxide as a promising, versatile, and sustainable choice for heterogeneous Fenton catalysis, making notable strides in addressing environmental challenges.

### 2.2. Copper Complexes/Nanoparticles

The advancement of heterogeneous catalysts that are efficient, environmentally friendly, and cost-effective based on copper for the remediation of wastewater is highly sought after and poses a notable challenge. Currently, the urgent global concern revolves around the exploration and adoption of novel and economical methods for catalytic oxidation, degradation, and removal of pollutants in wastewater and the environment. In the last few years, copper nanoparticles (Cu NPs)/complexes incorporated into polymers have been employed as effective catalysts for heterogeneous Fenton-like reactions. This is attributed to their characteristics, including a large surface area, thermal stability, and mechanical strength. These catalysts, whether composed of copper complexes or Cu NPs immobilized on polymers, can be acquired through diverse processes, encompassing methods involving ionic or metallic copper. As such, some researchers have pioneered the fabrication of copper catalysts on a range of polymers, such as polyethylene glycol [98], polyvinylpyrrolidone [99], polyampholyte [100], cellulose [101], and chitosan [57,102].

Chitosan hydrogel loaded with copper (Cu/CH) was developed as a catalyst for the decomposition of hydrogen peroxide through a straightforward method and utilized to generate ${HO}^{•}$ in a Fenton-like reaction [57]. The ${HO}^{•}$ concentration and the catalytic activity of Cu/CH were determined using the photoluminescence technique. Chitosan-supported copper can be recycled multiple times without a loss of its catalytic activity; this has been confirmed by reusability studies.

The catalytic uses of these heterogeneous catalysts in the elimination of contaminants are emphasized in this section. Thus, Orto et al. [100] noted the production and utilization of Cu(II)-polyampholyte as a robust catalyst for the degradation of methyl orange (MO) at room temperature through the activation of $H_{2}O_{2}$. The catalyst exhibited a degradation efficiency of over 90% for MO at pH 7.0 within 20 min. In contrast, the Cu(II)/$H_{2}O_{2}$ system without the polyampholyte resulted in the oxidation of less than 10% of the MO within the same period. This highlights the crucial role of the Cu(II)-polyampholyte in enhancing the catalytic performance, showing its potential for efficient pollutant degradation in environmental applications.

Castro et al. [103] prepared poly(4-vinyl pyridine)-supported Cu(II) for the oxidation of phenol using $H_{2}O_{2}$ as the oxidant. Poly(4-vinyl pyridine) (PVP) is an appealing choice for immobilizing metal ions because of the robust affinity of the pyridyl group towards metals and its ability to engage in hydrogen bonding. Examination of the PVP–copper complex’s behavior reveals that the presence of the carbonyl bond group is a function of the metal concentration [104]. Similarly, Lyu et al. [105] developed Cu-doped mesoporous polyimide nanocomposites (Cu-MP NCs) as catalysts for the Fenton-like process. In this case, the selected pollutant was the dye Rhodamine B (RhB), known for its high toxicity and biological resistance. In the presence of $H_{2}O_{2}$, only 30.8% and 39.8% of RhB were removed within 90 min using the traditional Fenton catalysts ${Fe}_{3}O_{4}$ and CuO (with the same metal content), respectively. RhB elimination was merely 26.5% in the polyimide Fenton-like system in the same conditions. Remarkably, in the Cu-MP NCs/$H_{2}O_{2}$ suspension, RhB elimination achieved 93.1% within just 30 min, and the pollutant was completely degraded at 60 min. This result was 28, 21, and 15 times higher than the removal rates in the suspensions of polyimide/$H_{2}O_{2}$, ${Fe}_{3}O_{4}$/$H_{2}O_{2}$, and CuO/$H_{2}O_{2}$, respectively. The reaction speed for pollutant elimination is 15–21 times greater than that of the conventional Fenton catalyst. Therefore, Cu-MP NCs exhibited high Fenton-like activity and efficiency for degrading refractory pollutants across a broad pH range.

**Figure 5:**
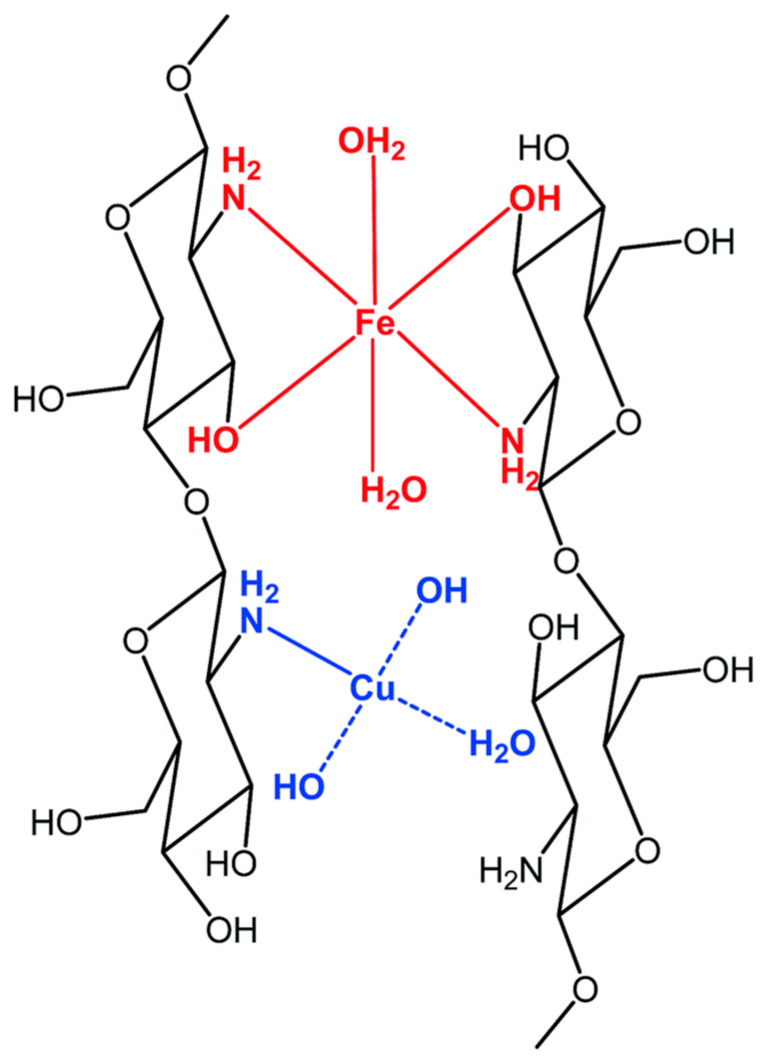
Configuration of the chitosan loaded with copper–iron bimetal complex Reproduced from [106]. Copyright 2011 with permission from Royal Society of Chemistry (London, UK).

The use of a bimetal catalyst is considered an alternative technique due to the synergetic action of the different metals present in the catalyst. In this sense, Shen et al. fabricated chitosan loaded with a copper–iron bimetal complex by chelating chitosan with ${Fe}^{3+}$ and ${Cu}^{2+}$ (Figure 5) [106]. The catalytic efficiency of the complex was evaluated in the degradation of RB 5, utilizing $H_{2}O_{2}$ as an oxidant within a pH range of 4–12. The catalyst effectively eliminated more than 90% of the dye within 10 min and demonstrated effective reusability and durability under the reaction conditions.

### 2.3. Ruthenium

Ruthenium (Ru) stands out as the sole member of the platinum group metals within transition metal catalysts displaying Fenton-like activity in the presence of $H_{2}O_{2}$ as an oxidant. Although the potential oxidation states of Ru span from 0 to +8, only the divalent (${Ru}^{2+}$), trivalent (${Ru}^{3+}$), and tetravalent (${Ru}^{4+}$) oxidation states are frequently encountered. A comprehensive study has been conducted on Ru complexes for different organic transformation reactions like alcohol dehydrogenation, olefin hydroxylation, water oxidation, and alkene epoxidation [107,108]. Nevertheless, there have been only a restricted number of studies published thus far on the removal of environmental contaminants employing ruthenium-mediated $H_{2}O_{2}$ decomposition [108]. The degradation of bisphenol A was accomplished by Hu et al. [109] employing a ${Ru}^{2+}$–polypyridyl complex immobilized on cation exchange resins (Dowex-50W and Chelex-100) as the catalyst. The reaction between ${Ru}^{2+}$ and $H_{2}O_{2}$ produced ${HO}^{•}$ within the pH range of 4 to 8, exhibiting improved oxidation efficiencies at higher pH levels. Similarly, a crosslinked pilar[5]arene polymer was synthesized via a click between a hydrophobic azide-modified pillar[5]arene and a hydrophilic alkyne-modified Ru derivative. The polymer, which was self-assembled into spherical nanoparticles with positively charged surfaces and catalytic ability, showed excellent performance in the removal of the anionic dye [110].

Employing resin as a polymer support not only prohibited the leaching of the Ru complex but also facilitated the repeated oxidation cycles and straightforward recovery of the catalyst. The latter is more important since Ru is an expensive and rare element. Therefore, the utilization of Ru-based Fenton systems in real-world applications might be restricted to situations that necessitate exceptionally robust catalytic activity and specific reaction conditions [107].

## 3. Synthesis Methods Employed in Preparing Polymer-Supported Heterogeneous Catalysts

The synthesis of polymer-supported catalysts such as metals or metal oxide NPs occurs through two main pathways: ex situ and in situ. The ex situ approach begins with the initial synthesis of inorganic NPs using soft chemistry routes, separately from the polymer matrix. These NPs are then dispersed in a three-dimensional matrix or a polymer solution. Ex situ synthesis provides precise control over the size, shape, and composition of the NPs before integrating them into the polymer support. Typical methods for the ex situ approach include chemical reduction, sol–gel methods, and thermal decomposition. This synthesis method is favored because it imposes no restrictions on the host polymers to be used and the choice of nanoparticles [111]. In addition to synthetic polymers, biopolymers like alginate and chitosan have been extensively employed for the ex situ synthesis of polymer-supported nanoparticles (PSNPs). Numerous biopolymers are soluble in acidified aqueous solutions. The creation of PSNPs containing a biopolymer typically entails conditioning the biopolymer in various physical forms and entrapping the NPs within these diverse shapes [112]. The procedure comprises two stages: polymer dissolution, and polymer neutralization, coagulation, or ionotropic gelation. This method has been extensively employed to produce spherical hydrogels. Table 2 summarizes several examples of methods employed for the synthesis of polymer-supported heterogeneous catalysts. 

In situ synthesis: within this approach the metal oxides or NPs of metal are synthesized within a pre-existing polymer framework or matrix. This is often achieved by adding metal precursors or reactants to the polymer solution or melt, followed by the initiation of nanoparticle formation through chemical or physical processes such as reduction reactions, precipitation, or thermal treatments. The combination of various functionalized polymers and different types of NPs that can be prepared using the in situ approach results in a wide range of possible PSNPs. In situ synthesis offers advantages such as improved NP dispersion within the polymer and reduced handling steps compared to ex situ methods. This method is becoming increasingly popular due to its technological advantages over ex situ methods, because particle size and morphology can be controlled with relative ease.

Several factors control the nature of the PSNPs such as the nature of the functional polymer, the composition of the metal and metal oxide NPs, the type of nanoparticle precursor and the reaction that forms the nanoparticles. In this procedure, the polymers act as nano-reactors, offering a confined medium for synthesis. They also secure and separate the produced NPs, averting their aggregation. While the interfaces among various synthesis methods are closely interlinked, the in situ process can be divided into two main groups: sorption, which is succeeded by a redox and/or precipitation reaction; and impregnation, which is followed by a precipitation and/or redox reaction.

Polymers with specifically designed functional groups, such as hydroxyl, carboxyl, or amine, are strategically developed to collaborate with metal ions. This promotes the adsorption of catalytic species, consequently improving the general effectiveness of the Fenton reaction [117]. Thus, polyacrylonitrile (PAN) contains nitrile groups that possess reactivity, allowing for their conversion into various functional derivatives. These functionalized polymers have application as heterogeneous catalysts [118]. Recently, Rubina et al. [118] prepared a hydrazine-modified PAN–iron complex that was reused in six successive cycles without iron leaching and loss of activity in MB degradation, showing the great potential of this green catalyst. The utilization of biodegradable polymers as supports for Fenton heterogeneous catalysts makes a positive contribution to the sustainability of the environmental remediation process of wastewater treatment [119]. The integration of biodegradable polymers in the Fenton reaction catalyst enhances the overall environmentally friendly character of the remediation process of wastewater treatment because these polymers can naturally decompose over time, reducing the long-term environmental footprint. This strategy demonstrates a deliberate endeavor to balance effective remediation with a dedication to environmental accountability and sustainability. Otherwise, polymers are integrated into membranes for water treatment, establishing a foundation for heterogeneous Fenton-like reactions to take place on the membrane surface, leading to the degradation of pollutants in water [120].

These versatile strategies underscore the importance of polymers in improving the performance and sustainability of heterogeneous Fenton-like processes for environmental remediation. Ongoing research explores novel polymeric materials and designs to optimize the efficiency of these catalysts in treating water contaminated with organic pollutants.

**Figure 6:**
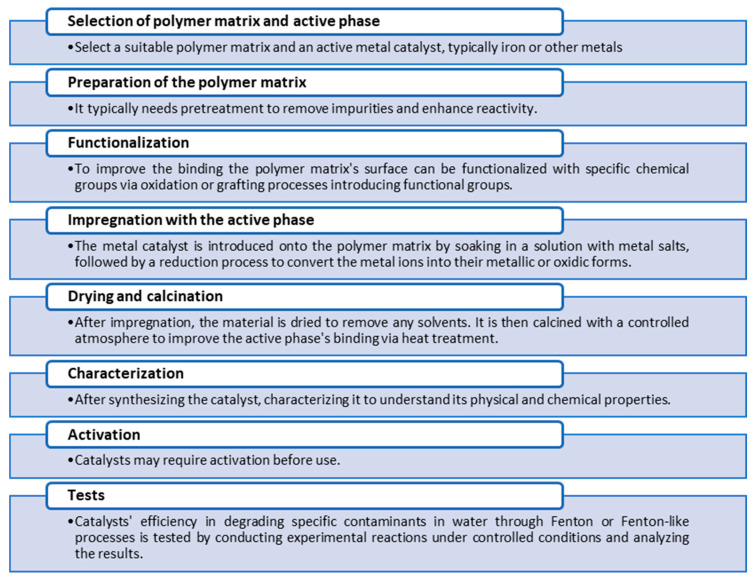
Steps for the synthesis of polymeric catalysts and before use in Fenton processes.

Based on the information recovered from the references, it is possible to generalize that the synthesis of polymeric catalysts before use in Fenton processes involves the steps shown in Figure 6. The specific details of the synthesis will vary significantly depending on the type of polymer catalyst and its intended use. The process may also involve optimization steps in which the reaction conditions, such as temperature, pH and metal loading, are adjusted.

## 4. Effect of Operational Parameters on the Fenton Process

It is necessary to pay special attention to operational factors because they play a principal role in shaping the characteristics of the polymer-supported heterogeneous catalyst employed in the process. Consequently, these parameters significantly impact the overall effectiveness of heterogeneous Fenton degradation. Proper control and optimization of operational parameters are essential for achieving desired outcomes and enhancing the efficiency of degradation reactions. The operating conditions to consider include catalyst dosage, pollutant concentration, and the pH value of the medium.

### 4.1. Catalyst Dosage

The catalyst dosage is a critical operational parameter in heterogeneous Fenton processes utilizing polymer-supported catalysts. Increasing the catalyst dosage typically supplies more active catalyst sites, accelerating the decomposition of $H_{2}O_{2}$. This, in turn, significantly increases the number of ${HO}^{•}$ radicals, leading to increased pollutant degradation rates [86]. Li et al. [96] pointed out that the degradation rate of TCs increased with an increase in the catalyst amount when the concentration of the catalyst was lower than 500 mg·L^−1^. After that, the elimination rate did not change significantly and even decreased as the catalyst amount increased further from 500 to 700 mg·L^−1^ [96]. When the catalyst dosage was 700 mg·L^−1^, the removal rate of TCs was lower than that at 500 mg·L^−1^. This phenomenon can be explained by several factors, including the increase in the catalyst dose, and the higher iron content resulting in an increase in the active sites, which corresponds to the variation in the efficiency of the degradation of contaminants. However, with a further increase in the catalyst dosage, a substantial formation of ${HO}^{•}$ occurs. As the highly active ${HO}^{•}$ radicals have a very short lifetime, a considerable number of them start to quench rapidly due to the swift reactions between ${HO}^{•}$ [121]. Consequently, the degradation efficiency decreases (Equation (4)).

(4) $${HO}^{•}+{HO}^{•}\;\;\to \;H_{2}O_{2}$$

### 4.2. Pollutant Concentration

The effect of the pollutant concentration in heterogeneous Fenton processes is a key determinant of the effectiveness of the approach. The concentration of contaminants directly affects several aspects, such as reaction kinetics [118]. The removal efficiency of MB was found to decline slowly with an increase in the initial MB concentration [119]. Nevertheless, the elimination rates of MB at 15, 20, and 30 mg L^−1^ were 46.41, 42.47, and 23.30%, respectively. As the concentration of MB increased, the duration of the degradation process extended. This occurred because the quantity of ${HO}^{•}$ produced in the reaction system became constant when the doses of the carboxylate-rich carbon (CRC)-modified ${Fe}_{3}O_{4}$ magnetic particles (CRC/${Fe}_{3}O_{4}$) catalyst and $H_{2}O_{2}$ were constant. At lower contaminant concentrations, the ${HO}^{•}$ radicals in the solution are relatively abundant. Nevertheless, as the quantity of pollutants expands, the ${HO}^{•}$ generated in the solution should be relatively insufficient. This makes it necessary to extend the reaction time to effectively eliminate the higher concentration of the contaminants solution.

In addition, the concentration of pollutants can affect the pathways and mechanisms of the Fenton reaction [122]. Different concentrations might result in different reaction pathways, resulting in the formation of diverse by-products. At higher pollutant concentrations, the possibility of unintended reactions and the generation of more by-products increases. This is because there is a greater abundance of pollutants available for reaction with ${HO}^{•}$, leading to more complex chemical transformations.

### 4.3. Effect of pH

Determining the optimal pH range plays an essential role in heterogeneous Fenton-like reactions, to achieve a desirable efficiency in the process of wastewater treatment. Nevertheless, the optimal pH range remains a subject of controversy among researchers, given the varying opinions about its range. In light of various investigations into heterogeneous Fenton-like reactions at neutral pH, or under alkaline conditions, effective elimination of organic pollutants can be achieved [90], whilst other research illustrated that a pH around 3 is suitable [123]. The primary reason for this could arise from the variable solubility of metal ions and the diverse activities of active sites. Certain catalysts could be utilized essentially by the active sites on their surface, while through the release of metal ions from the catalyst surface, other varieties have the potential to function as catalysts. In the initial case, the catalytic function is carried out by the active sites; so, they may exhibit significant resistance to pH influences. On the other hand, for the latter case, due to the hydrolysis and the precipitation, an increase in pH could effectively deactivate the metal ions in the aqueous phase and potentially delay or prevent the leaching of metal ions from the solid catalyst surface.

In light of different studies and their findings, an optimal pH point in heterogeneous Fenton-like processes in wastewater remediation always exists. The optimal pH value represents a balance between preventing composite degradation, promoting radical generation, and ensuring effective electrostatic attraction between the pollutant and the catalyst. Nevertheless, it should be noted that the use of polymers with pH-responsive characteristics as coatings for heterogeneous Fenton catalysts ensures the continued effectiveness of the catalyst at the specific pH conditions essential to achieve the highest Fenton-type reaction efficiency [124].

## 5. Recent Developments in Polymer-Supported Heterogeneous Fenton Catalysts

**Figure 7:**
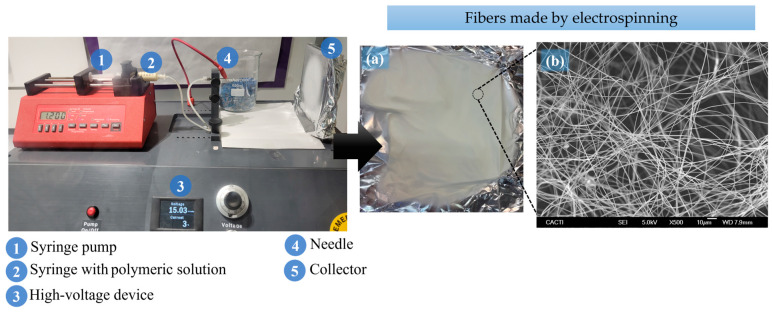
Electrospinning equipment and photos of (**a**) PAN fiber on aluminum foil and (**b**) scanning electron microscope. Adapted from [125]. Copyright 2024 with permission from MDPI (Basel, Switzerland).

Recent techniques for the synthesis of polymer-based catalysts for Fenton reactions have focused on improving the dispersion of active metal sites and increasing the stability and efficiency of the catalyst. Among them, the use of electrospinning technology to produce polymeric membranes has received special interest among the scientific community due to its versatility and potential applications. Electrospinning is an adaptable technique that allows the generation of polymeric fibers from submicrometer to nanometer diameters by applying an electrical charge (Figure 7) [125]. This method makes it possible to control the morphology, diameter, pore distribution and surface area of the fibers, which are essential for catalytic applications [126].

Thus, the interest in the electrospinning technique for obtaining catalytic membranes lies in its ability to produce highly porous and fibrous structures with a high surface area/volume ratio. This morphology improves mass transfer and provides abundant active sites for catalytic reactions [127]. In addition, electrospinning allows precise control over the composition, structure, and properties of the membranes, which allows them to be adapted to specific catalytic applications. In addition, the technique offers scalability and versatility, which makes it suitable for various catalyst support materials and configurations. When applied to the creation of membranes that serve as catalysts in Fenton processes, electrospinning offers the following advantages [128,129]:

- High specific surface area: Electrospun membranes have a high specific surface area, which provides more active sites for catalysis and increases reaction efficiency.
- Controllable porosity: The diameter and porosity of the fibers can be adjusted during the electrospinning process, which allows for optimized adsorption of contaminants and diffusion of reagents.
- Improved stability and reduced iron leaching: Since the iron ions are immobilized within the polymer matrix instead of being free in solution, the formation of iron hydroxide sludge is avoided, which is a significant advantage over homogeneous Fenton systems.
- Reusability: Membranes produced by electrospinning can be used multiple times with sustained efficiency, reducing operating costs.

Another additional advantage is that these polymeric membranes can be easily functionalized with compounds such as triethanolamine. Thus, a heterogeneous Fenton catalyst was produced by electrospinning polyurethane membranes functionalized with triethanolamine and the iron was loaded, doping FeCl_3_ on the membrane. During the electrospinning process, parameters such as needle-to-collector distance, voltage, flow rate, and spinning time are carefully controlled to achieve the desired fiber thickness and membrane porosity [130]. These polyurethane-based synthesized membranes offer several advantages. They demonstrate high catalytic efficiency in the decomposition of common wastewater contaminants such as chromium, MB, and MO. Their physical properties allow for easy recovery and reuse, making them cost-effective in practical applications. It is important to highlight that in the functionalization with triethanolamine, the membrane exhibited changes. Thus, the electrospun polyurethane without functionalization showed a fiber diameter of 2.2 μm that decreased to 1.3 μm by the incorporation of triethanolamine and FeCl_3_. This reduction in fiber diameter can be attributed to the increased conductivity of the polymer solution after the addition of triethanolamine [131], resulting in a higher charge density of the droplet formed at the needle tip, and thus, a longer jet. The improved porous structure, compared to the bare polyurethane membrane, provided more catalytic sites for reactions.

Recently, the integration in the polymeric membrane of metal–organic frameworks (MOFs) for heterogeneous catalyst materials has been explored [132]. Recently, the use of dual or bimetallic MOFs exhibited higher performance with more active sites and higher charge transfer capacity compared to the individual components. Thus, Fdez-Sanromán [133] developed a one-pot synthesis of a bimetallic Fe–Cu MOF composite with high removal of dyes, drugs, and pathogens.

In all cases, these membranes maintain significant degradation performance over multiple cycles, indicating good stability and potential for repeated use. However, there are challenges associated with the electrospinning process, including the need for precise control of spinning parameters and the potential complexities involved in scaling up for industrial applications. In addition, environmental and economic factors must be considered when evaluating the long-term feasibility and effectiveness of electrospun membranes as Fenton catalysts in wastewater treatment.

Another recent technique is self-polymerization confinement, which is designed to address some of the major challenges such as the increasing of the metal loading and maintaining a high dispersion of metal nanoparticles while preventing agglomeration and leaching. This process requires a carbonization stage in which the polymeric network of metal ions undergoes a controlled pyrolysis process at high temperatures in an inert atmosphere to give rise to a nitrogen-doped carbon matrix containing the metal NPs. The final material may undergo further activation steps, such as acid washes or heat treatments, to increase its porosity and expose more active metal sites [134]. Along these lines, Wang et al. [135] obtained ultrafine metallic NPs embedded within a nitrogen-doped carbon matrix by a process involving the self-polymerization of dopamine around the metal ions. This created a confined space in which polymerization could occur, anchoring the metallic NPs in place and resulting in highly charged and well-dispersed metal–nitrogen–carbon catalysts.

## 6. Conclusions and Final Remarks

Current pollutants necessitate increased attention since the prevalence, destiny, and surveillance of these pollutants are not universally understood. The current imperative is to alert researchers to the existence of these compounds in minute concentrations within water and wastewater treatment facilities. Among the current significant concerns is the presence of these contaminants, prompting the need for their removal through a sustainable method. For this reason, advocating the integration of heterogeneous Fenton-like catalytic systems is underscored as an ideal approach. Their noteworthy adaptability allows them to adapt smoothly to diverse water treatment challenges, ensuring an effective response to different emerging pollutants. The dependability of these catalytic systems further strengthens their endorsement, instilling confidence in their capability to steadily tackle and neutralize contaminants in wastewater. Furthermore, their reusability not only aligns with sustainable practices but also presents a practical and economical solution for the ongoing elimination of emerging pollutants from wastewater.

The many-sidedness of the polymer-supported heterogeneous Fenton catalyst has been convincingly demonstrated by numerous researchers, showcasing its effectiveness in removing a wide range of organic pollutants. The constraints associated with iron catalysts, especially the tendency to aggregation and the low adsorption capacity, can be overcome by the incorporation of catalysts on a polymer support. Polymeric materials can provide support for a heterogeneous Fenton catalyst while also enhancing the effectiveness of Fenton reactions. Numerous advancements have occurred in polymer-supported heterogeneous Fenton catalysts. First, the enhanced catalytic performance, a focus of researchers who are working on optimizing the composition, structure, and properties of polymer-supported materials. Second, the novel polymer matrices that innovations are exploring offer improved stability, better reusability, and increased compatibility with Fenton reaction conditions. Third, the functionalization, since functional groups on polymer matrices are introduced to enhance the affinity for Fenton-active species, leading to improved catalytic performance.

Despite the ongoing research developments, they remain insufficient to confirm the effective elimination of many contaminants identified in wastewater. Therefore, numerous contaminants require increased attention. To thoroughly examine the presence and future consequences of substances of increasing concern in the environment, a thorough investigation is essential. Additionally, enhancing the efficiency of heterogeneous Fenton processes may be achieved by connecting external energy sources, such as light, microwave, electricity, and ultrasound, with the Fenton-like reaction. The goal is to optimize the catalytic performance, leading to enhanced remediation of wastewater.

Finally, when selecting the best polymer matrix and/or metal–polymer composite for Fenton-like decomposition of pollutants, two critical factors to consider are compatibility and recyclability. Ensure compatibility between the polymer support and metal or metal oxide NPs to prevent leaching or degradation, which could affect the catalyst’s effectiveness and cause environmental harm. Additionally, design composites that facilitate easy separation and recycling of catalysts to reduce waste generation and improve sustainability. This approach promotes a more environmentally friendly and economically viable process. Therefore, further studies should be undertaken in the future focused on verifying the efficiency of the processes and their implementation in real wastewater treatment, as well as evaluating the scaling and operation strategies in flow systems, aspects that are fundamental for the applicability of processes based on the Fenton reaction.

## Figures and Tables

**Table 1:** Presents the effectiveness of iron oxide/polymer mixed composites in heterogeneous Fenton elimination of pollutants.

Metal/or Metal Oxide	Polymer Matrix	Pollutant	Removal (%)	Ref.
Ferrous ions	Chitosan	MB	99% (30 min)	[44]
${Fe}_{3}O_{4}$	PEDOT	RB5	90% (10 min)	[53]
${Fe}_{3}O_{4}$	Pullulan hydrogels	Tetracycline hydrochloride	91.36% (180 min)	[55]
${Fe}_{3}O_{4}$	Poly(catechol)	MB	100% (120 min)	[89]
${Fe}_{3}O_{4}$	Chitosan	Tetracyclines	96.0% (20 min)	[90]
Goethite	PVA-alginate	1-butyl pyridinium chloride	100% (60 min)	[91]
α-Fe_2_O_3_	PVA	Tetracycline	100% (60 min)	[92]

**Table 2:** Summarizes several examples of the methods employed for the synthesis of polymer-supported heterogeneous catalysts.

NP	Polymer Designation	Procedure of Fabrication	Ref.
${Fe}_{3}O_{4}$ (magnetite)	Carboxylated polyacrylamide	NPs were combined with N,N-methylenebisacrylamide and potassium peroxydisulphate before the addition of acrylamide monomers. Following polymerization, the resulting PSNPs were functionalized using succinic anhydride dissolved in dioxane at pH 4.	[113]
Fe/Fe oxide	Poly(methylmethacrylate) (PMMA)	A suspension of NPs and PMMA in acetone was spin-cast and dried to produce magnetically active films.	[114]
Magnetite	Chitosan and alginate	Chitosan with ${CaCl}_{2}$ was blended with NPs before introducing an alginate solution to create hydrogels, which were subsequently dried for use as an adsorbent.	[115]
${Fe}^{3+}$/${Ni}^{2+}$ oxides and hydroxides	Alginate	NPs were blended with an acidified alginate solution and subsequently introduced into a calcium chloride solution.	[116]
${Fe}_{3}O_{4}$	Poly(3,4-ethylenedioxythiophene)	Magnetite NPs in PVA were combined with 3,4-ethylene-dioxythiophene, followed by the addition of HCl. The acid exposes some ${Fe}^{3+}$ ions, initiating polymerization and catalyzing the formation of PSNPs.	[53]

## Data Availability

Not applicable.
